# AI Through Ethical Lenses: A Discourse Analysis of Guidelines for AI in Healthcare

**DOI:** 10.1007/s11948-024-00486-0

**Published:** 2024-06-04

**Authors:** Laura Arbelaez Ossa, Stephen R. Milford, Michael Rost, Anja K. Leist, David M. Shaw, Bernice S. Elger

**Affiliations:** 1 Institute for Biomedical Ethics, University of Basel, Basel, Switzerland; 2 Institute for Research on Socio-Economic Inequality (IRSEI) in the Department of Social Sciences, University of Luxembourg, Esch-Sur-Alzette, Luxembourg; 3 Care and Public Health Research Institute, Maastricht University, Maastricht, The Netherlands; 4 Center for Legal Medicine (CURML), University of Geneva, Geneva, Switzerland

**Keywords:** Artificial intelligence, Healthcare, AI guidelines, Guidelines, Ethics, AI ethics, Regulatory affairs, Regulations

## Abstract

While the technologies that enable Artificial Intelligence (AI) continue to advance rapidly, there are increasing promises regarding AI’s beneficial outputs and concerns about the challenges of human–computer interaction in healthcare. To address these concerns, institutions have increasingly resorted to publishing AI guidelines for healthcare, aiming to align AI with ethical practices. However, guidelines as a form of written language can be analyzed to recognize the reciprocal links between its textual communication and underlying societal ideas. From this perspective, we conducted a discourse analysis to understand how these guidelines construct, articulate, and frame ethics for AI in healthcare. We included eight guidelines and identified three prevalent and interwoven discourses: (1) AI is unavoidable and desirable; (2) AI needs to be guided with (some forms of) principles (3) trust in AI is instrumental and primary. These discourses signal an over-spillage of technical ideals to AI ethics, such as over-optimism and resulting hyper-criticism. This research provides insights into the underlying ideas present in AI guidelines and how guidelines influence the practice and alignment of AI with ethical, legal, and societal values expected to shape AI in healthcare.

**Supplementary Information:**

The online version contains supplementary material available at 10.1007/s11948-024-00486-0.

## Introduction

The increasing number of Artificial intelligence (AI) ethics guidelines reflects the growing recognition of AI’s potential benefits and risks. As AI technology advances, there is increasing enthusiasm for AI, especially machine learning (ML) techniques, because of their capacity to analyze already available health data for preventive, diagnostic, or treatment support (Leist et al., 2022). However, the assumption that AI applications might become more prevalent in society has raised concerns over the ethical implications of its use. Common questions include what is necessary to trust AI, respect people's autonomy, and avoid biases and discrimination (Floridi et al., 2018; Murphy et al., 2021). AI guidelines aim to guide our approach to AI for the benefit of society through the use of principles, statements, rules, or recommendations. As such, academic, (non)governmental, and other institutions worldwide have published guidelines to guide AI development and those working with it.

Reviews of generic AI guidelines (AI used across settings without specific healthcare focus) have sought to map and examine the common themes and areas of focus they address (Bélisle-Pipon et al., 2022; Fjeld et al., 2020; Fukuda-Parr & Gibbons, 2021; Jobin et al., 2019; Ryan & Stahl, 2020). Some concerns generic AI guidelines address include privacy, bias, transparency, autonomy, explainability, well-being promotion, and responsibility. These reviews provide a helpful overview of the state of AI ethics guidelines to understand critical issues and challenges related to AI ethics. Although generic AI guidelines could apply across different disciplines, some guidelines specifically address the use of AI in healthcare. These guidelines strongly emphasize considering the ethical implications of using AI in medical decision-making and other healthcare applications. A prominent example is the World Health Organization (WHO) publication on "ethics and governance of artificial intelligence for health" (World Health Organization, 2021).

The field of AI in healthcare is still relatively new, and there is an ongoing debate about the best approaches to ensuring the ethical use of AI. Noticeably, the use of AI in healthcare raises specific ethical issues related to the beneficence and respect of autonomy, as patients and communities require assurance that introducing AI would not jeopardize their rights. Beyond challenges inherent to AI, decisions taken in healthcare are frequently intertwined with high-risk scenarios and highly sensitive data. Health is central to individual well-being; doctors must support, safeguard, and advocate for patients. For example, an essential pillar of medical ethics, shared decision-making between patients and their doctors, could be affected by the introduction of AI as a potential threat to patients' and doctors' autonomy if AI does not account for their rights and preferences (Abbasgholizadeh Rahimi et al., 2022).

Guidelines as a form of written language can be analyzed to identify the links between textual communication and our societal ideas. Discourse (i.e. a group of ideas or patterned ways of thinking in textual form) not only reflects but reproduces our social realities with its dominant beliefs, power structures, and ideologies (Lupton, 1992). Discourse analysis (DA) as a qualitative methodology can analyze the contextual structure surrounding communication, including the context in which it takes place and how it shapes a common sociocultural understanding (Fairclough, 2022; Lupton, 1992; Yazdannik et al., 2017). From that perspective, the discourse in ethical guidelines for AI can significantly shape the healthcare community's understanding and approach to ethics. Therefore, guidelines discourse requires particular attention because it is a powerful driver for discussing and (re-)orienting AI ethics. For example, guidelines can base their ideals on practical (e.g., efficiency), technical (e.g., performance), or ethical (e.g., beneficence) frameworks, thus, helping to legitimize certain foundations, concepts, and notions in AI ethics for healthcare. Therefore, AI guidelines can establish a common framework for thinking about and addressing ethical issues in AI. In that sense, it is essential to look at the understanding of ethics in AI guidelines and critically examine if it meets the moral requirements of healthcare settings.

This paper analyzes how guidelines construct, articulate, and frame AI ethics for healthcare. The aim is to look beyond what is written and critically interpret these guidelines' underlying ideologies ((Cheek, 2004; Lupton, 1992; Yazdannik et al., 2017). As such, we are interested in how the guidelines shape AI ethics in healthcare, including whose perspectives are considered when determining ethical issues in AI and the implications for ethics, AI, and healthcare stakeholders.

## Methods

Previous work has synthesized generic AI guidelines through thematic or content summaries (Fjeld et al., 2020; Jobin et al., 2019; Ryan & Stahl, 2020). Policy and social researchers have used Critical Discourse Analysis (CDA) to understand public health documents, albeit this methodology has yet to be applied to AI-guiding documents. However, the usability of CDA has been visible in other domains, for example, by using CDA to examine how health policy documents constructed chronically ill patients' roles or how inclusion policies framed health inequalities (Tweed et al., 2022; Walton & Lazzaro-Salazar, 2016). Other researchers used CDA to analyze the discourse surrounding AI in social media and the academic discussion on artificial general intelligence (Graham, 2022; Mao & Shi-Kupfer, 2021; Singler, 2020). Given the importance of written AI guidelines for understanding AI ethics for healthcare, we undertook a CDA of AI guidelines, which allows us to have an in-depth interpretation of the construction, articulation, and framing of AI ethics for healthcare. Therefore, we aimed to analyze the discourse in AI guidelines rather than systematically map the content and themes.

### Identifying Relevant Studies

First, given the absence of a unified database for AI healthcare guidelines, we reviewed all the documents inventoried by previous researchers for potential inclusion. Additionally, we reviewed database initiatives that track AI policies: Nesta’s^1^ “AI governance database”, Algorithm Watch’s^2^ “AI Ethics Guidelines Global Inventory”, OECD.AI’s^3^ “policy observatory”, and AI Ethics Lab’s^4^ “Toolbox: Dynamics of AI Principles”. We use a purposive sample to find documents written by influential institutions such as governments, intergovernmental organizations, or non-profit organizations. Second, Google Search was used as a general search engine because AI guidelines are not academic publications and thus fall under the "gray literature" category. The first author searched and screened for AI guidelines to select a final set.

### Inclusion and Exclusion Criteria

For this review, we consider ‘AI guidelines’ to be documents that provide ethical guidance, including policies, guidelines, principles, or position papers introduced by governmental, inter-governmental, or professional organizations. Including this type of AI guidelines allow us to analyze how influential institutions construct, articulate, and frame AI ethics in healthcare. To be included, guidelines must provide normative guidance for AI in healthcare: principles, tenets, recommendations, propositions, or tangible steps for developing or implementing AI in healthcare.

We excluded documents that provided observations regarding advances in AI for a particular year. Additionally, we excluded “internal” company principles due to the limited intended audience, as they are primarily created for the respective institution. We also excluded documents solely focusing on one disease application or a specific medical specialty because these might not be generalizable to other healthcare contexts. We finalized the search in August 2022. The first author screened 179 document titles. We excluded 169 documents because they either did not qualify as guidelines or were outside the scope of this review (i.e., documents that were not about AI or were unrelated to healthcare). Summary of reasons for exclusion in Supplementary materials 2.

### Analysis

We departed from the analytical positivist approach of a systematic literature review.^5^ DA is a diverse methodology for analyzing the language in use and how discourse creates a shared understanding of a topic. DA goes beyond the content of words and interprets how a topic is constructed, represented, and reflected within its context (Fairclough, 2013, 2022). In particular, we used CDA because language expresses and shapes social and political relationships, and its analysis can uncover underlying ideologies or power dynamics.

We transferred the guideline texts to a qualitative data management software (MAXQDA) to carry out data analysis. We analyzed the guidelines in three phases. First, the first author read the included guidelines in detail and extracted high-level information. During data familiarization, the authors discussed preliminary ideas on trends in the guidelines and created a list of specific questions that we considered relevant to answer the main research question. The first author analyzed the guidelines in the second phase by creating high-level analytical themes that focus on organizing the material into the following discourse strands: How do guidelines (1) discuss ethical motivation to develop and implement AI and ethics (e.g., what is the justification and primary goal of guidelines); (2) construct ethical AI (e.g., if guidelines used principles); (3) assign the roles of different stakeholders. Third, all authors tested and critically interrogated the analytical themes and organization of results. The authors reached a consensus about the structure and characteristics of the several discourses. This process eventually resulted in the description of three discourses.

## Results

See Fig. 1.

**Fig. 1:**
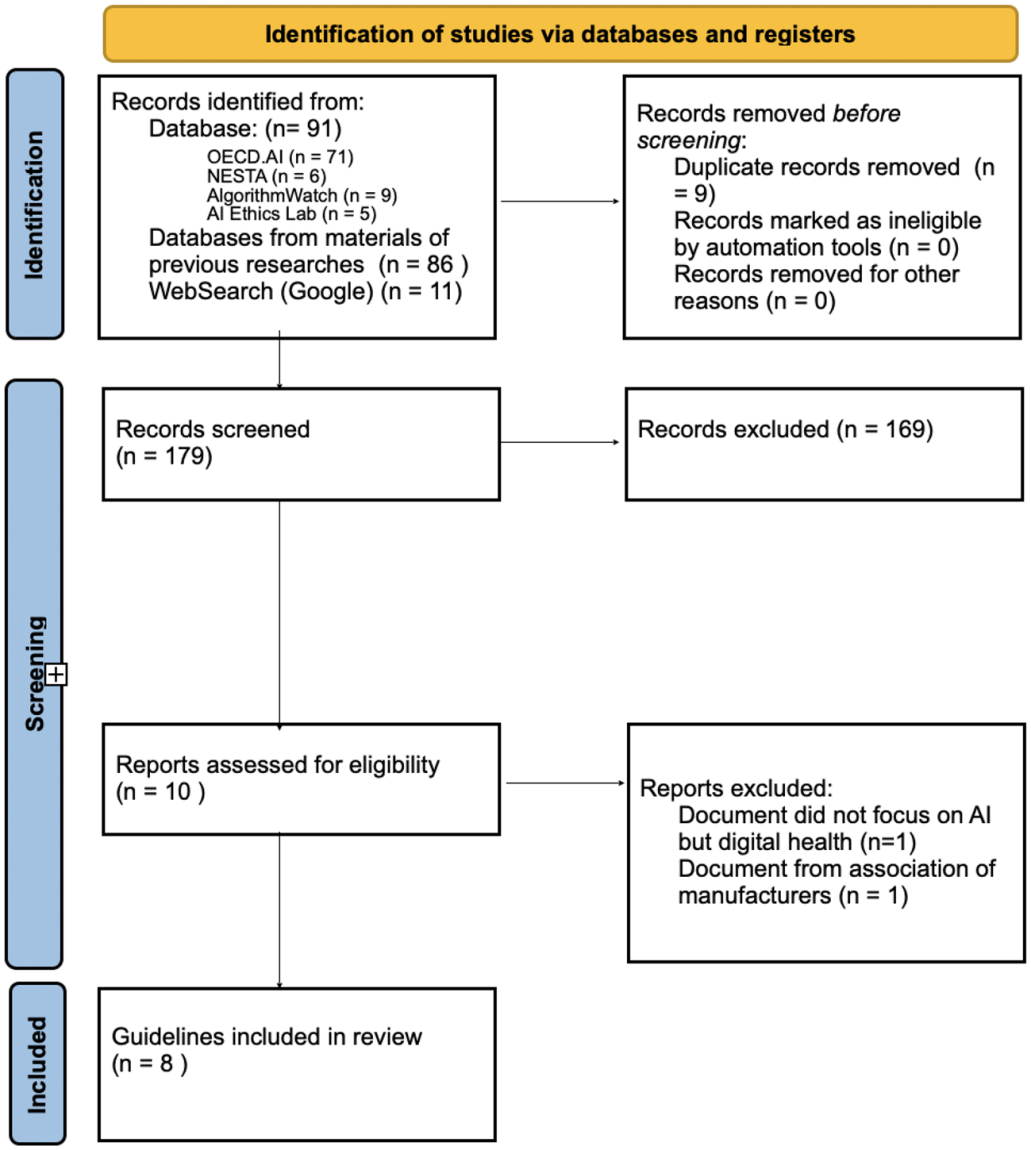
Flow diagram (PRISMA) (Page et al., 2021)

Applying the selection criteria led to eight guidelines ultimately being included in this analysis (Supplementary materials 1). Most of them were published in 2021. Intergovernmental organizations published two documents. All other guidelines came from high-income countries (the United Kingdom, the United States of America, Canada, Singapore, and the United Arab Emirates) (Table 1). The length of the documents varies widely, with G1 being the longest (114 pages) and G5 the shortest (two pages). Guidelines G3, G5, G6, and G7 focus on (general) good practice or good AI. Guidelines G2, G4, and G8 are generally intended to guide AI in healthcare but do not specifically focus on ethical AI. Guideline G1 focuses on ethics and governance.

**Table 1:** Description of included documents

No.	Name	Issuing body	Type of document	Retrieval	Year
G1	Ethics and governance of artificial intelligence for health	WHO	Guidelines	Google Search	2021
G2	Trustworthy AI in Health	OECD, G20 dialogue	Input for discussion	OECD.AI	2020
G2.1	Recommendation of the Council on Artificial Intelligence	OECD/Legal/0449	Recommendations	snowball from document 2	2019
G3	A guide to good practice for digital and data-driven health technologies	UK government	Guidelines	Through OECD.AI (indirect from an initiative), AI Ethics Lab and Nesta	2021
G4	Policy on the Use of AI in the Healthcare Sector	United Arab Emirates	Policy	Nesta	2018
G5	Good Machine Learning Practice for Medical Device Development: Guiding Principles	U.S. Food and Drug Administration (FDA), Health Canada, and the United Kingdom’s Medicines and Healthcare products Regulatory Agency (MHRA)	Guiding principles	Snowball from FDA results on the OECD.AI	2021
G6	Deliverable 1: principles for the evaluation of artificial intelligence or machine learning-enabled medical devices to assure safety, effectiveness and ethicality	UK government	Policy	Google Search	2021
G7	Deliverable 2: principles to support the development and deployment of artificial intelligence or machine learning-enabled medical devices across jurisdictions	UK government	Policy	Snowball from Deliverable 1 (previous document)	2021
G8	Artificial intelligence guidelines in healthcare	Singapore	Guidelines	Google Search	2021

### Audience

The guidelines address AI developers (G1, G8) but also describe them as innovators (G3, G6), and manufacturers (G4, G7, G8). Other described addressees are policymakers (G1, G2, G5), healthcare professionals (G1, G4) and healthcare institutions (G1, G4). "AI actors" describes all stakeholders in the AI system lifecycle (G2.1 p. 7). The guideline G8 uses an umbrella group called 'implementers' that could include healthcare professionals and institutions. To this extent, G8 acknowledges that the "*groups are not mutually exclusive*" (G8 p. 8), which creates some uncertainties in interpreting guidelines for individual stakeholders. The guidelines sometimes discuss AI recommendations without specifying a responsible party. For example, G4 mentions the need for verifiable and explainable AI without indicating who should ensure this (G4 p.8). Guideline G5 mentions a human in the loop without describing anyone specifically.

## Lack of standard definition of AI

Most guidelines focus their discussion on AI (G1, G2, G4, G6, G7). Four guidelines make a distinction: G3 describes “*digital and data-driven technologies*” that include AI, guideline G5 focuses only on machine learning (ML), and G6-G7 combines both as AI/ML-enabled medical devices (Supplementary materials 3 in Table 1).

The guidelines lack a standard definition of AI, thus, leading to different interpretations between data-driven programs (such as prediction or diagnosis) and a potential program that resembles a more general state of intelligence (human-like cognition). When the object of regulation is still a topic of debate, it may result in regulating entirely different or not yet existing systems, including Artificial General Intelligence. Consequently, these guidelines could evoke an understanding of AI driven by the potential human-like capacities of the systems rather than a more measurable technical definition. Informing the definition of AI with such futuristic perceptions may contribute to the mystification of AI and increase fears regarding its application. Fears can result in learned helplessness, where people disengage from AI, diminish participation in discussions, and become relegated to passive acceptance and hindering participation (Lindebaum et al., 2020).

### Discourse 1: AI is Unavoidable and Desirable

All guidelines agree that AI will be an agent of change in medicine. Discussions on AI are fundamentally based on its potential, making these AI guidelines future-looking, prospective, and, to some extent, speculative. Most guidelines describe the benefits and risks of AI techniques (G1, G2, G3, G5, G6, G7, G8). For example, G2 states that AI in healthcare has *"profound potential, but real risks*"(G2 p. 7). The guideline G5 mentions that AI and ML “*have the potential to transform health care […], but they also present unique considerations due to their complexity and the iterative and data-driven nature*” (G5 p. 1). In doing so, guidelines frequently juxtapose opportunities and threats while justifying the need for considerations to avoid harm. Therefore, guidelines tend to describe their primary motivation as avoiding harm while harnessing the promised potential of AI technologies (Supplementary materials 3 in Table 2). These statements are pragmatic formulations derived from the (unspoken) assumption that AI will be implemented and that healthcare needs to make the best of it. However, this type of discourse entails a matrix of beliefs: AI is an unavoidable development and undeniably useful.

**Table 2:** Examples of discussions regarding patient-centric and patients’ roles

No	Patient-centricity and comments regarding patients’ role
G1	*“Patients, community organizations and civil society should be able to hold governments and companies to account, to participate in the design of technologies and rules, to develop new standards and approaches and to demand and seek transparency to meet their own needs as well as those of their communities and health systems”.* (G1, Executive Summary, p. xvi) *In relation to patients’ engagement: “the public should be engaged in the development of AI for health in order to understand forms of data sharing and use, to comment on the forms of AI that are socially and culturally acceptable and to fully express their concerns and expectations”* (G1 p. 71)
G3	*People need to know that their data is being used for their benefit and that their privacy and rights are safeguarded”* (G3 p. 5) *“An appropriate Disaster Recovery and Business Continuity Plan should be in place. This should be explicit in covering how any risk to patient data and, more importantly, patient health, will be limited and mitigated”* (G3 p. 10)
G6	Foster a patient-centred approach: “*We acknowledge the importance of patient and public voices in the development and deployment of AI/ML-enabled medical devices in health. We intend to provide assurance that AI/ML-enabled medical devices are co-designed with stakeholders from diverse backgrounds.*”(G6 p. 7)
G8	Patient-Centricity: “*Safeguards in the design, development, and implementation of* [Artificial intelligence] *should be put in place to ensure that patients’ interests, including their safety and well-being, are protected.*” (G8 p. 7) *“implementers of* [Artificial intelligence] *must continue to comply with all applicable laws and requirements (*etc*.), including the seeking of appropriate patient consent”. “Consent required when using* [Artificial intelligence] *should be no different from consent taken for other medical procedures performed by actual physicians”* (G8 p. 33)

Guidelines fail to be sufficiently cautionary against the techno-cultural ideals and the hype surrounding technological developments. The pressure to adopt innovation based on enthusiasm and economic or technical forces could undermine the debate about demonstrating that AI improves healthcare quality (Dixon-Woods et al., 2011). Guideline G1 (G2 also, to some extent) questions whether AI should be used (or not) and the risk of overestimating the benefits of AI or dismissing the risks (G1 p. 31–33). None of the guidelines were sufficiently critical against the base assumptions that AI is an agent of benefits and progress in medicine. However, there is no evidence yet of this change because most AI systems are not currently used in daily real clinical scenarios. For example, a guideline states that they *“recognizes that AI holds great promise for the practice of public health and medicine”* (G1 p. xi). The guideline G6 states that “*the use of AI/ML […] presents a significant potential benefit to patients and health systems in a wide range of clinical applications […]*” (G6 p. 4). In that sense, there is an unspoken but present assumption that AI is mainly—at least potentially—beneficial and that if used correctly, AI will change life and medicine. In the guidelines, the desire to harness or guide the potentials of AI indicates that this innovation is at least an acceptable reality or a potentially desirable development. This discourse might echo sentiments from the technology industry, where innovation is the ultimate goal and something new might be better just because it is new. However, a strong pro-innovation stance could lead to risk-taking or scientifically unfounded experimentation for innovation and change. Slota et al. rightly pointed out this challenge and have critically questioned that innovation may not be positive per se and cannot be unquestionably accepted and suggested that innovation needs to abide by prerequisites to be considered positive, for example, reliability measurements (Slota et al., 2021).

When guidelines base their discussion primarily on AI’s potential, AI might have a special status compared to other healthcare innovations, especially because AI’s potential became a justification for its support and development. For example, drug development guidelines request manufacturers to establish benefit/risk assessment on the evidence for a drug’s safety and effectiveness to improve, change or remove diseases. Guidelines are cautious, even when using unproven interventions (with no evidence available through clinical trials), emphasizing that potential benefits must be substantial and that there should be no other alternatives (EMA, 2018a; FDA, 2019). Giving AI special treatment due to the desire to realize AI’s potential risks prompts technology companies to take advantage of their expertise and unduly influence governmental decisions regarding AI’s regulation and practices. For example, in contact tracing technology for Covid-19 (although not always AI-enabled), government concerns over data privacy allowed technology companies to gain influence because of their expertise in data privacy, inadvertently permitting them to influence how this technology was developed (Sharon, 2021). As technology develops, many small decisions need to be taken, which, when combined, can significantly impact how a policy is implemented and its practical interpretation. In AI guidelines, industry representatives are often involved and may have an imbalance of influence over the development of these guidelines compared with other directly impacted stakeholders such as patients (Bélisle-Pipon et al., 2022).

### Discourse 2: The Necessity of Principles to Guide AI

Despite using different terms, having different aims, and addressing different stakeholder groups, guidelines agree that AI needs principles to be guided. However, there is wide variation in the usage and conceptualization of these principles, with most documents not clarifying the theoretical basis for including them. Only G1 provides an account of their definition of principles, which references bioethics and human rights as the theoretical framework. G6–G7 cross-references the definition and construction used in G1. There is no common assumption about the conceptual framework behind using these principles, leaving their interpretation and operationalization up to the reader's discretion (Supplementary materials 3 in Table 3).

Positively, guidelines aim to help AI be developed within the acceptable limits of society and human ideals, including safety protocols. However, the guidelines see principles as a viable, feasible, and acceptable solution to guide AI. This cultural understanding could have originated from the influential science fiction work by Isaac Asimov, in which robots must follow hardwired social and moral norms (do no harm to humans, obey humans, and protect themselves) (Asimov, 1950; Jung, 2018). Asimov’s laws were the author’s answer to finding protection against the potential malicious consequences of technology, though he also acknowledged in his work the potential for conflict between these laws. Using principles in the guidelines comes from a similar perspective whereby there are concerns about the potential negative consequences of AI.

Guidelines fluctuate between discussions on important principles and how to apply these and develop acceptable AI. For example, G6–G7 discusses aspects of AI such as suitability and robustness while adding ethical aspects such as inclusiveness, fairness, or risks for health discrimination. Guideline G1 starts with ethical principles and continues to add recommendations on AI’s development, while G8 includes fairness in the guiding principles and recommendations for data representativeness. The guideline G3 requests manufacturers to ensure “t*he product is easy to use and accessible to all users*” and “*ensure that the product is clinically safe to use*” which are both operationalizations (G3 p. 7, 9). The same guideline (G3) also asks manufacturers for ethical behavior and to “*be fair, transparent and accountable about what data is being used*” (G3 p. 12). Although more technical, several guidelines (G5, G6, G7, G8) do not provide measurable estimations on AI’s behavior or what is acceptable. For example, stating that “*to promote technical robustness, manufacturers […] should test performance by comparing it to existing benchmarks, ensuring that the results are reproducible […]and reported using standard performance metrics*” (G6 p. 13). However, there is no mention of what would be acceptable for performance metrics or how to select acceptable benchmarks.

Most guidelines emphasize *"non-maleficence"* (G1, G2, G3, G4, G6, G7, G8). However, the emphasis on producing no harm could create a paradoxical interpretation where ‘no harm’ becomes the aim. For example, G1 discusses its principle to “*promote human well-being, safety and public interes*t” by stating that “*AI technologies should not harm people. They should satisfy regulatory requirements for safety, accuracy, and efficacy […] to assess whether they have any detrimental impact […]. Preventing harm requires that use of AI technologies does not result in any mental or physical harm*” (G1 p. 26). These prevention-framed messages emphasize behavior to avoid possible negative consequences. Still, they do not highlight what benefits can justify the usage of AI. Moreover, avoiding all harm might be an unrealistic expectation for AI. For example, an AI robot that performs surgery needs to produce an injury (surgical incision) to perform a procedure. If the principles aim to avoid all physical harm, would it be acceptable to have a surgical AI? In the discourse, it is difficult to clarify. Moreover, patients' risk acceptance is not a dichotomous ‘all or nothing,’ as most patients understand that risk is a spectrum of likelihood. For example, patients with psoriasis were willing to accept the risk of serious infection between 20 to 59% as a side effect of their treatment, depending on their disease severity (Kauf et al., 2015). There are nuances in what is acceptable for healthcare stakeholders, and creating principles—although appealing—might not meet healthcare needs. Hutler et al. utilize a similar example of a surgical robot to state that it is not as simple as “training” robots to avoid harm and that challenges exist to conceptualize what is harmful and what should be morally allowed while designing robots (Hutler et al., 2023).

Nearly all guidelines consider transparency or explainability essential for ethical, good, or responsible AI (G1, G2, G4, G5, G6, G7, G8). However, explainability is a debated concept without consensus on its importance or meaning (Mittelstadt et al., 2019). Guidelines often see transparency as an enabler of ethical practices by rendering AI’s processes visible and able to be held accountable (unclear if AI or the people working with it). However, there is no unified definition or acceptability about what and when AI is transparent. Considering that an explainable AI equals ethical AI might be a fig leaf where AI developers cover methodological shortfalls by providing end-users with a false understanding (Starke et al., 2022). In contrast, when these principles aim to provide a basis for technical assurance, they should be described as technically feasible and operationalizable. In the current form, guidelines principles seem to be best followed as a thought experiment that re-analyzes the expectations for AI rather than a static set of rules for AI’s development or ethical behavior.

### Discourse 3: The Primacy of Trust

Guidelines frame trust, as in ‘*trustworthy AI*’, as the answer to overcoming public doubt. While well-performing AI might build trust, when the center of the discussion is on trustworthy AI, there is a shift from performance expectations (quality) to trust. Reading statements within the guidelines in which trust is central gives one the impression that trust matters more than AI's usability, feasibility, or performance. For example, G1 acknowledges that "*trust is key to facilitating the adoption of AI in medicine*." (G1 p. 48); G2 discusses entirely trustworthy AI, and G6-G7 repeatedly discusses trustworthy innovation. Guideline G3 mentions that achieving algorithm transparency can “*build trust in users and enable better adoption and uptake*” (G3 p. 16). Potentially, these statements implicitly apply trustworthiness as a quality seal for good AI, although trust and good are slippery concepts and do not equate to one another. For example, a guideline mentions that “*discussions are crucial to guide the development and use of trustworthy AI for the wider good”* (G2 p. 6). The guideline G3 states, *“we must approach the adoption of these promising technologies responsibly and in a way that is conducive to public trust,*” (G3 p. 5). Some guidelines consider the lack of trust to impede the usage of data. For example, a guideline mentions that *“lack of trust […], in how data are used and protected is a major impediment to data use,and sharing.”* (G2 p. 16). Others equate trust as an impediment to the development of AI itself; for example, mentioning “*whether AI can advance […] depends on numerous factors beyond the state of AI science and on the trust of providers, patients, and health-care professionals*” (G1 p. 15). These arguments frame trust as a commodity (measured, managed, or acquired) for the benefit of innovation or technical interests instead of focusing on the preconditions for acceptable AI, such as technical robustness, proven effectiveness, and protection frameworks in case of errors (Krüger & Wilson, 2022).

When guidelines describe trust as a means to further innovation, they may fall into the role of advocates for technology, especially when they motivate or suggest that trust in AI is crucial. For example, a guideline “re*cognizes that ethics guidance […] is critical to build trust in these technologies to guard against negative or erosive effects and to avoid the proliferation of contradictory guidelines*” (G1 p. 3). The guideline G8 states that “*with the increasing use of healthcare AI […], the intent of the [guideline] is to improve clinical and public trust in the technology by providing a set of recommendations to encourage the safe development and implementation[…]*” (G8 p. 5). This discourse indicates that (1) public trust in AI matters; (2) there might be concerns that the public does not trust AI. The importance of healthcare stakeholders, especially patients, is narrowed to the expectation of acquiring their trust and their position of vulnerability in healthcare.

Patients’ roles are discussed concerning data protection, safety assurance, and as subjects that must trust AI. There is a cursory mention of "*patient-centricity*" in the guidelines and the importance of patients in AI design. Guideline G1 mentions the importance of patients and their role in ensuring "*human warranty*". Guideline G3 mentions that patients need assurance, G4 mentions patients as part of their potential audience. Although these guidelines touch on other situations requiring patients' input, they do not give them an active voice. Most guidelines focus on informing patients about AI (G1, G3, G6, G7, G8) and their data usage. Guidelines discuss the role of patients as subjects worthy of protection due to their vulnerability in healthcare but limit their role to passive bystanders (Table 2). Guidelines have tended to focus more on treating patients as mere data subjects. While G1, G5, G8 mention a citizen participation mechanism as they welcome feedback through public docket or direct contact, the feedback is only collected after the first iteration of guidelines. None of the guidelines are written specifically for patients, by or in collaboration with patients, even though guidelines advocate for including patients in AI’s design. In generic AI ethics guidelines, researchers observed that the lack of stakeholder engagement is a prevalent issue, with less than 6% included citizen participation (Bélisle-Pipon et al., 2022). Most guidelines do not mention allowing patients to decide if or when to use AI. Uniquely among the guidelines, G8 refers to patients’ ability to decide whether to continue using AI or receive care from a clinician instead (G8 p. 33). Another guideline, for example, only allows people “*to opt out of their confidential patient information being used for purposes beyond their individual care and treatment*” (G3 p. 13).

## Discussion

Our analysis of guidelines for AI in healthcare identified a lack of a standard definition of AI and three main discourses: (1) AI is a desirable and unavoidable development, (2) Principles are the solution to guiding AI, and (3) Trust has a central role. Important for the intended audience of these documents (mainly software developers, but also innovators and manufacturers) is that the discourses were largely concerned with AI applications possibly available in an undefined future. Each of the guidelines discourses cannot be taken in isolation as, to some extent, they reference and influence each other. For example, G1 references the definitions used in G2, and G6-G7 references the principles in G1. In that sense, there may be certain reproductions of ideas that do not exclusively represent the vision of the publishing institution. While acknowledging this possibility, in its totality, the discourses seem to be, in many instances, determined by broader societal discourses, such as the technology industry's optimistic and innovation-driven ideals. In a review of techno-optimism, Danaher concludes that while common in industry and policy, strong forms of techno-optimism may be unwarranted without further analysis and justifications (Danaher, 2022). However, the optimistic assessment of AI regarding its qualities and faculties is well-established in other policy documents for generic AI applications. In a discourse analysis published after the completion of this paper, researchers reviewed policies from China, the United States, France, and Germany that also established AI as inevitable and framed an interdependence between technology and societal good creating a powerful rhetoric “that sheds pivotal attention and necessity to AI, lifting it into a sublime aura of a savior”(Bareis & Katzenbach, 2022). In the broad European policy context—albeit also not healthcare specific—researchers found that AI is also represented as a “transformational force, either with redeeming of “salvific” qualities drawing from techno-solutionist discourse, or through mystified lens with allusion to dystopian narratives” (Gonzalez Torres et al., 2023). Our results demonstrate that similar discourses are built into the AI guidelines for healthcare.

While experts and institutions contributing to the guidelines have made a commendable effort to stay on top of AI innovation, the guidelines are undoubtedly a work in progress. In particular, the discourses show a tension between a pro-growth stance (AI as medical progress) and the need for caution (guidance, principles, trust, and ethics). For example, technical performance metrics, such as achieving the highest accuracy in prediction or classification, can conflict with ethical performance, which aims to avoid making decisions based on sensitive attributes or proxies of those attributes. The problem is already part of the discussion in non-AI clinical decision algorithms where race has been (wrongly) used to change risk assessments, for example, kidney function (Vyas et al., 2020). For the current AI discussion, it is unclear how to reconcile both views and if we can or should. For example, commitments to ensuring AI is fair or respects human dignity might not be specific enough to be action-guiding or operationalizable. On the contrary, focusing simply on technical measurements could not meet ethical requirements. Cybersecurity and data protection are often conflated with respect for autonomy or non-maleficence, potentially simplifying the interpretation and applicability of the ethical value. Ethically, respect for autonomy is associated with the right of patients to decide if, when, and how to receive health care. Operationalizing respect for autonomy would include a discussion on patients’ consent to use AI, including their preferences, and not only about data consent. To an extent, AI ethics might fail to uphold its boundaries, especially to the techno-optimism driving AI and its techno-solutionism.

Most guidelines do not include the sociotechnical context of those involved in AI. The most common addressees (developers, innovators, and manufacturers) might need a more comprehensive understanding of ethical concepts during their training or support afterward. Indeed, some ethical statements in these guidelines are meaningless without the proper ethical acculturation. For example, ethical education in computer science degrees in Europe is often a standalone subject with limited hours (Stavrakakis et al., 2022). The discourse often addresses stakeholders' responsibilities (using terms such as ‘should’ or ‘shall’). However, there is limited engagement in defining rights. For example, what are the rights of end-users? The rights could be implicit, but if the desire is to promote the active engagement of other non-technology stakeholders in the ethical development of AI, they should be made aware of their rights and educated about their options.

As an overarching analysis, we identified that AI guidelines switch between technical and ethical expectations, concepts, and notions. Other applications are precise in distinguishing their aim and intended usage. For example, guidance for medical devices for cervical cancer includes quality management, standards, and operational consideration (WHO, 2020). As another example, good manufacturing practices describe the minimum standards pharmaceutical manufacturers must meet in their production processes (EMA, 2018b; European Commission, 2003; WHO, 2014). Quality-by-design is an approach to ensure the quality of medicines by *“employing statistical, analytical and risk-management methodology in the design, development, and manufacturing of medicines”* (EMA, 2018c). Finally, Good Clinical Research Practice (GCP) principles are descriptive and focus on making research scientifically sound and justifiable (WHO, 2005). The lack of precision could be one of the reasons why there has been a backlash against utilizing ethics as a framework to inform AI guidelines. Some academics have criticized AI ethics for being toothless, useless or vague (Fukuda-Parr & Gibbons, 2021; Héder, 2020; Heilinger, 2022; Munn, 2022). Critics have mentioned that AI ethical guidelines do not offer robust strategies to protect human rights and cannot emphasize accountability, participation, and remedy as protection mechanisms for people (Fukuda-Parr & Gibbons, 2021). Others have criticized AI ethics in its current form, for the difficulty of implementing moral ideals in technological practices and the lack of consensus on ethical principles for AI (Munn, 2022).

The criticism of AI ethics might be due to a misconception of the role of ethics and the way guidelines are constructed, articulated, and framed for healthcare. Simplifying guidelines as a document that includes all AI, aims to guide in all scenarios, and tries to cover all stakeholders is over-ambitious. Compared to guidelines in other medical areas, principles for AI include autonomy, transparency, non-maleficence, fairness, trust, and responsibility. Therefore, AI’s approach to ethics tends to remain abstract, hindering the value of AI ethics and its potential application (Zhou & Chen, 2022). For example, ethics-by-design in AI replaced quality-by-design in pharmaceutical development. Ethics-by-design aims to make people consider ethical concerns and requirements such as respect for human agency, privacy and data governance, transparency, fairness, and individual, social, and environmental well-being (European Commission, 2021). However, ethics-by-design is not as operable as quality-by-design. When the goal is to operationalize ethics, AI guidelines might lack qualitative and quantitative suggestions to validate when and how to achieve and respect the proposed principles (Zhou & Chen, 2022). Therefore, limiting the contribution of AI ethics and potentially legitimizing content-thin ethics that are easy—at least pretend to be easy—to follow. In that sense, the criticism of ethical guidelines does not directly signal a failure of ethics but a potential over-spill between theoretical boundaries and aims. In the worst case, these guidelines can delay effective legislation. Guidelines can be used for ethics-washing, where it becomes easier to appear ethical than take ethical actions, especially if guidelines rely on forms of self-regulation and there are no legal consequences for the actions or if the content of the guidelines is abstract or general (Wagner, 2018). AI actors could use superficial recommendations as a red herring, resulting in widely ignored or superficially followed guidelines because they lack operational consequences for their choices.

## Limitations

To our knowledge, this is the first comprehensive review of healthcare AI guidelines (from governments or institutions) from an ethical perspective, carried out by a multidisciplinary team. Although including various subjects (bioethics, philosophy, medicine, public health, theology, and psychology), our background has certainly informed our research and influenced our analysis. However, to overcome these challenges, we have reflected on our positionality and analyzed the guidelines in a nonlinear nature that forced us to contest our assumptions continuously. Given the continuous development of AI guidelines, the vast nature of AI, and our available resources, we noted several limitations. We did not aim to do a systematic review but to examine the widely available and influential guidelines worldwide critically. However, some relevant documents might have been excluded because they are hard to locate online or unavailable in the public domain. Limiting the analysis to English documents implied some linguistic exclusions and might limit a broad geographical interpretation. The search ended in the first half of 2022, which might be too early as most of the included guidelines were published from 2021 onwards. For example, the WHO outlined considerations for regulating artificial intelligence for health in Nov 2023, which indicates that other guidelines may be available since the final completion of this paper in Feb 2023. At least two research teams have done discourse analysis of AI policies, and have been published recently—albeit not healthcare-specific (Bareis & Katzenbach, 2022; Gonzalez Torres et al., 2023). The search for gray literature is challenging and could lead to biased inclusion of those documents which contain key search terms in their titles. We could not include guidelines from Latin America, Central Asia, or Africa, as none of the available guidelines fulfilled the inclusion criteria (domain-specific guidelines for healthcare). Previous researchers have acknowledged this limitation because they have also been unable to analyze guidelines from those geographical regions (Jobin et al., 2019). However, we noticed that initiatives are starting to emerge for the general governance of AI, such as national strategies (Kenya) or data focus AI guidelines in several Latin American countries (Gaffley et al., 2022; tmg, 2020). Given the nature of CDA as a qualitative research method, our results cannot be generalized for other guidelines not included in this study.

## Conclusions

While AI systems may be required to adhere to existing legal frameworks, it may be necessary to modify or augment these frameworks to account for the unique considerations posed by AI. These guidelines will inform other forms of regulations, and it is vital to understand what they establish throughout their discourse (Larsson, 2020). It is essential that guidelines clarify their intentions and that they stand, at least as much as possible, immune to undue influence from the technology industry. Currently, guidelines tend to be over-enthusiastic about the capacities of the technology and the possibilities of change. First, AI is a broad concept, and guiding the development of something general is challenging. Second, it is dangerous to consider everything through the lenses of potential (benefits and risks). Like technology, AI ethics can be a victim of hype and reduce credibility. Third, supposing the concepts and conceptualizations employed in the guidelines are not thoughtfully considered. In that case, there is a risk that the guidelines may endorse values that fail to align with the needs of society. Guidelines focus on analyzing the potential benefits and risks of having an all-smart AI while focusing less on the social context necessary to use AI ethically. For example, except for G1, most guidelines do not explicitly address the fact that some public health problems could be equally—or less expensively—addressed via non-technical solutions. Similarly to guidance for pharmaceutical development, guidelines could recommend a justification to use technology, either because there are no better options or when it is demonstrably the best strategy.

Future AI guidelines for healthcare could benefit from implementing other approaches if they wish to guide ethical development. For example, patients' limited contribution could be resolved using participatory strategies such as citizen advisory groups. Other approaches beyond principles could be pertinent to achieving the goals of AI ethics. The Swiss Medical Association (FMH) issued practical demands for the development of AI instead of principles: defining AI's role as a medical device, requesting AI to follow evidence-based medicine practices, and assigning doctors and patients roles as coordinators of care (FMH, 2022). Defining AI and people’s roles in the form of ‘usage requirements’ could be another way to achieve the objective of integrating AI in healthcare. Care ethics focuses on relationships, dependencies, and societal and cultural factors that could help contextualize AI solutions to their intended application. Alternatively, process-based ethical frameworks are a valid basis because AI is not a single solution or a single problem. Also, other approaches, such as codes of conduct for specific stakeholders, might bring the expected results of guiding the people working with AI. For example, a code of conduct would be more useful if they address specific stakeholders as it can go in-depth and analyze role-based problems. The construction of AI ethics guidelines in its current form is narrow, focusing on creating or identifying a static list of principles and not engaging in more thorough approaches. A change would require an awareness of the potential of ethics as a framework for moral inquiry and a deep understanding of the purpose of AI ethics and its limits. Future guidelines iterations, therefore, might need to refine, shift and reshape their approach to AI guidelines and AI ethics.

### Supplementary Information

Below is the link to the electronic supplementary material.

Supplementary file1 (PDF 75 kb)

Supplementary file2 (DOCX 13 kb)

## Footnotes

1 Nesta is a UK based agency for social good. There developed a pilot project to map global initiatives for AI governance https://www.nesta.org.uk/data-visualisation-and-interactive/mapping-ai-governance/

2 AlgorithmWatch is a non-profit research and advocacy organization that is committed to watch, unpack and analyze automated decision-making (ADM) systems and their impact on society.https://algorithmwatch.org/en/

3 Global policy observatory by the OECD https://oecd.ai/

4 AI Ethics Lab aims to detect and address ethics risks and opportunities in building and using AI systems to enhance technology development. https://aiethicslab.com/big-picture/

5 The positivism paradigm aims to obtain explanations and predictions by relying on hypothetico-deductive method to verify a priori hypotheses that are often stated quantitatively (Park et al., 2020). In contrast, DA methodologies focus on interpreting data in a social context.
