# Supplementary material for: AI Through Ethical Lenses: A Discourse Analysis of Guidelines for AI in Healthcare
Source: Sci Eng Ethics. 2024 Jun 4;30(3):24. doi: 10.1007/s11948-024-00486-0 (PMC11150179; doi:10.1007/s11948-024-00486-0)
Supplement: Supplementary file 1 — Supplementary file1 (PDF 75 kb) [file 11948_2024_486_MOESM1_ESM.pdf]

AI Through Ethical Lenses: a Discourse Analysis of Guidelines for AI in Healthcare

Authors:

Arbelaez Ossa, L[1] , Milford S.R.[1] , Rost M[1] , Leist A.J.[2] , Shaw D.M.[1 3] , Elger B.S.[1 4]

[1]Institute for Biomedical Ethics, University of Basel, Basel, Switzerland.
[2] Institute for Research on Socio-Economic Inequality (IRSEI) in the Department of Social Sciences, University of Luxembourg, Esch-sur-Alzette, Luxembourg.

[3] Care and Public Health Research Institute, Maastricht University, Netherlands.

[4] Center for Legal Medicine (CURML), University of Geneva, Switzerland.

Corresponding author:

Laura Arbelaez Ossa, laura.arbelaezossa@unibas.ch

Annex 1: Summary of included guidelines

Guideline No.	Title, Publisher (Year)	Authors (working group)	Type of Guidance	Principles
G1	Ethics and governance of artificial intelligence for health. WHO (2021)	WHO worked with 20 experts (from: Jordan, Japan, Tunisia, Canada, USA, Netherlands, Singapore, Uganda, India, South Africa, Denmark, UK, Italy, Chile, Switzerland, New Zealand, and China).	Principles and recommendations	1. Protect autonomy<br>2. Promote human well-being, safety, and public interest<br>3. Ensure transparency, explainability, and intelligibility<br>4. Foster responsibility and accountability<br>5. Ensure inclusiveness and equity<br>6. Promote AI that is responsive and sustainable

G2	TRUSTWORTHY AI IN HEALTH. OECD , G20 dialogue (2020).	Organization of Economic Co/operation and Development's Directorate for Employment, Labour and Social Affairs, and Directorate for Science, Technology, and Innovation.<br>(Note: authors stated that this document doesn't represent the view of OECD or the G20).	Principles and policy recommendations	Same as G2.1
G2.1	Recommendations on the Council for Artificial Intelligence . OECD/LEGAL/0449 (2019)	Secretary-General of the OECD.	Principles	1. Inclusive growth, sustainable development and well-being<br>2. Human-centered values and fairness<br>3. Transparency and explainability<br>4. Robustness, security, and safety<br>5. Accountability
G3	A guide to good practice for digital and data-driven health technologies. UK government (2021).	UK government	Principles and recommendations	1. How to operate ethically<br>2. Have a clear value propostion<br>3. Usability and accessibility<br>4. Technical assurance<br>5. Clinical safety<br>6. Data protection<br>7. Data transparency<br>8.Cybersecurity<br>9. Regulation<br>10. Interoperability and open standards<br>11. Generate evidence that the product achieves clinical, social, economic or behavioural benefits<br>12. Define the commercial strategy
G4	Policy on the Use of AI in the Healthcare Sector. United Arab Emirates (2018).	Department of Health UAE	Principles	1. Transparency<br>2. User Assistance / Supportive technology<br>3. Safety and Security<br>4. Privacy<br>5. Ethics<br>6. Accountability

G5	Good Machine Learning Practice for Medical Device Development: Guiding Principles. U.S. Food and Drug Administration (FDA), Health Canada, and the United Kingdom's Medicines and Healthcare products Regulatory Agency (MHRA) (2021).	U.S. Food and Drug Administration (FDA), Health Canada, and the United Kingdom's Medicines and Healthcare products Regulatory Agency (MHRA)	Principles	1. Multi-Disciplinary Expertise Is Leveraged Throughout the Total Product Life Cycle<br>2. Good Software Engineering and Security Practices Are Implemented<br>3. Clinical Study Participants and Data Sets Are Representative of the Intended Patient Population<br>4. Training Data Sets Are Independent of Test Sets<br>5. Selected Reference Datasets Are Based Upon Best Available Methods<br>6. Model Design Is Tailored to the Available Data and Reflects the Intended Use of the Device<br>7. Focus Is Placed on the Performance of the Human-AI Team<br>8. Testing Demonstrates Device Performance During Clinically Relevant Conditions<br>9. Users Are Provided with Clear, Essential Information<br>10. Deployed Models Are Monitored for Performance and Re-training Risks Are Managed
G6	Deliverable 1: principles for the evaluation of artificial intelligence or machine learning-enabled medical devices to assure safety, effectiveness and ethicality. UK government (2021).	UK government	Principles and recommendations	1. Champion inclusiveness, fairness, and transparency<br>2. Foster a patient-centred approach<br>3. Provide proportionate continuous evaluation<br><br>Key principles for Clinical Evaluation<br>1. Assessing the suitability of the AI/ML-enabled medical devices to the need<br>2. Evaluating the technical performance of the AI/ML-enabled medical devices<br>3. Evaluating the clinical performance of the AI/ML-enabled medical devices<br>4. Evaluating the long-term safety and wider impact of AI/ML-enabled medical devices

				5. Evaluating ethical aspects of AI/ML-enabled medical devices, including inclusion and fairness
G7	Deliverable 2: principles to support the development and deployment of artificial intelligence or machine learning-enabled medical devices across jurisdictions. UK government (2021).	UK government	Principles and recommendations	1. Champion inclusiveness, fairness, and transparency<br>2. Foster a patient-centred approach<br>3. Provide proportionate continuous evaluation<br><br>Principles to support the development<br>1. Understanding the data<br>2. Understanding the model<br>3. Robustness
G8	Artificial intelligence guidelines in healthcare.Singapore (2021).	Ministry of Health Singapore, Health Science Authority, Integrated Health Information Systems.Endorsed by: Academy of Medicine Singapore, College of Family Physicians Singapore, Infocomm Media Development Authority, Personal Data Protection Commission Singapore.	Principles and recommendations	1. Fairness<br>2. Responsibility<br>3. Transparency<br>4. Explainability<br>5. Patient-Centricity
