# Supplementary material for: AI Through Ethical Lenses: A Discourse Analysis of Guidelines for AI in Healthcare
Source: Sci Eng Ethics. 2024 Jun 4;30(3):24. doi: 10.1007/s11948-024-00486-0 (PMC11150179; doi:10.1007/s11948-024-00486-0)
Supplement: Supplementary file 2 — Supplementary file2 (DOCX 13 kb) [file 11948_2024_486_MOESM2_ESM.docx]

**AI Through Ethical Lenses: a Discourse Analysis of Guidelines for AI in Healthcare**

# **Authors:**

Arbelaez Ossa, L¹ , Milford S.R.¹ , Rost M¹ , Leist A.J.^2^ , Shaw D.M.¹ ^3^ , Elger B.S.¹ ^4^

¹Institute for Biomedical Ethics, University of Basel, Basel, Switzerland.

^2^ Institute for Research on Socio-Economic Inequality (IRSEI) in the Department of Social Sciences, University of Luxembourg, Esch-sur-Alzette, Luxembourg.

^3^ Care and Public Health Research Institute, Maastricht University, Netherlands.

^4^ Center for Legal Medicine (CURML), University of Geneva, Switzerland.

# **Corresponding author:**

Laura Arbelaez Ossa, [laura.arbelaezossa@unibas.ch](mailto:laura.arbelaezossa@unibas.ch)

# **Annex 3: Tables with data extracts from guidelines**

## Table 1: Conceptualizations on AI and ML

| **Guideline No.** | **Conceptualizations on AI** | **Conceptualizations on ML** |
| --- | --- | --- |
| G1 | *“Generally refers to the performance by computer programs of tasks that are commonly associated with intelligent beings”* (G1 p. 4).  Also referenced the definition of document 2.1 | *“Machine learning, which is a subset of AI techniques, is based on use of statistical and mathematical modelling techniques to define and analyse data. Such learned patterns are then applied to perform or guide certain tasks and make predictions”.* (G1 p. 4) |
| G2-G2.1 | *“An AI system is a machine-based system that can, for a given set of human-defined objectives, make predictions, recommendations, or decisions influencing real or virtual environments. AI systems are designed to operate with varying levels of autonomy”.* (G 2.1 p. 7) |  |
| G3 | AI is part of “*data-driven technologies*”. This guideline references another document [(Joshi & Morley, 2019 p.15)](https://www.zotero.org/google-docs/?97XYJU)  The referenced document explains that AI is difficult to define and describes four different definitions. |  |
| G4 | *“The mimicking of human thought and cognitive processes to solve complex problems automatically”* (G4 p. 6) |  |
| G5 | *“Artificial intelligence and machine learning technologies [...] use software algorithms to learn from real-world use and in some situations may use this information to improve the product’s performance”.* (G5 p. 1) | |
| G6-G7 | Uses AI/ML. These guidelines use the definition proposed by the International Medical Device Regulation Forum.  *“AI a branch of computer science, statistics and engineering that uses algorithms or models to perform tasks and exhibit behaviours such as learning, making decisions and making predictions”* (G6 p. 5) | *“Machine learning (ML) is described as a subset of AI that: allows computer algorithms to learn through data, without being explicitly programmed, to perform a task”.* (G6 p. 5) |
| G8 | *“An (AI medical device) AI-MD as defined by the Health Sciences Authority (HSA) refers to AI solutions which are intended to be used for investigation, detection, diagnosis, monitoring, treatment or management of any medical condition, disease, anatomy or physiological process. AI-MDs typically have a direct impact to patient safety”.* ( G8 p. 5) |  |

## Table 2: Examples of benefits/risks discourse

| **Guideline**  **No.** | **Benefits juxtaposed with risks** |
| --- | --- |
| G1 | *"Ethics guidance based on the shared perspectives of the different entities that develop, use or oversee such technologies is critical to build trust in these technologies, to guard against negative or erosive effects and to avoid the proliferation of contradictory guidelines”.* (G1 p. 3)  *“There are also potential serious negative consequences if ethical principles and human rights obligations are not prioritized by those who fund, design, regulate or use AI technologies for health.”* (G1 p. xi) |
| G2 | *“This paper discusses the promises and perils of AI in health, and the key policy questions that policymakers will need to address in an uncertain landscape. [...] so as to effectively manage the risks while not unnecessarily limiting the opportunities”.* (G2 p. 4) |
| G3 | *“Furthermore, increasing use of data-driven technologies, including artificial intelligence (AI), could cause unintended harm if we do not think about issues such as transparency, accountability, safety, efficacy, explicability, fairness, equity and bias”.* (G3 p. 5) |
| G4 | *“Seeks to maximize the efficiency, effectiveness, quality, sustainability and access-related benefits gained from using AI in healthcare services and minimize any potential risks to safety, outcome, and experience”* (G4 p. 8) |
| G6-G7 | *“Regulators, international standards bodies and health technology assessors across the world are grappling with how they can provide assurance that AI/ML-enabled medical devices are safe, effective and performant – not just under test conditions but in the real world”*  (G6 p. 5) |
| G8 | *“To improve clinical and public trust in the technology by: a. Providing a set of recommendations to encourage the safe development and implementation of primarily AI-Medical Devices [...]”.* (G8 p. 5)  *“These guidelines aim to share good practices with the healthcare community to guide the safe development of AI in healthcare”* (G8 p. 4) |

##

## Table 3: Guidelines reference principles.

| **No.** | **Principles Conceptualizations** | **Based on:** |
| --- | --- | --- |
| G1 | ***Definition****: “An ethical principle is a* ***s****tatement of a duty or a responsibility in the context of the development, deployment and continuing assessment of AI technologies for health.”* (G1 p. 23)  ***Intended use:*** *“These ethical principles are intended to provide guidance to stakeholders about how basic moral requirements should direct or constrain their decisions and actions in the specific context of developing, deploying and assessing the performance of AI technologies for health. These principles are also intended to emphasize issues that arise from the use of a technology that could alter relations of moral significance”* ( G1 p. 23) | Human rights frameworks and bioethics big 4 principles (non-maleficence, beneficence, justice, autonomy) |
| G2-G2.1 | ***Definition****:* not available  ***Intended use****: “The G20 AI Principles provide a framework to guide the discussion. Their value-based principles aim to foster innovation and trust in AI by promoting the responsible stewardship of trustworthy AI while ensuring respect for human rights and democratic values.”* (G2 p. 15)  *“Promote and implement the following principles for responsible stewardship of trustworthy AI, which are relevant to all stakeholders.”* (G2.1 p. 7) | Not clearly described |
| G3 | ***Definition****:* not available  ***Intended use****: “These principles of good practice can be built into the strategy and product development ‘by design’. This, in turn, will mean that when products are presented for assessment or procurement, many of the criteria in the specification will have already been met. The intention is to smooth the path between development and procurement so that the NHS may realise the benefits that digital technologies can bring”* (G3 p. 4) | Not clearly described However, “how to operate ethically” is the first principle for good practice |
| G4 | ***Definition****:* not available  ***Intended use****: “Guiding principles” with the vision of “To encourage the use of AI to enhance the reach, performance and precision of healthcare related services and minimize any potential risks to patient safety.” and the goal of “To encourage the safe and secure use of AI in healthcare management”* (G4 p. 7) | Not clearly described |
| G5 | ***Definition****:* not available  ***Intended use****: “These guiding principles will help promote safe, effective, and high-quality medical devices that use artificial intelligence and machine learning (AI/ML).” (G5 p. 1)* | Not clearly described |
| G6-G7 | ***Definition****: “We follow the WHO’s key ethical principles for the use of AI for health [...], to define the scope of what we mean by ethical. The breadth of this definition goes beyond the remit of medical device regulators.”* (G6 p. 6)  ***Intended use****: “Key principles for clinical evaluation [...] enabled medical devices for healthcare to* *protect the safety of patients across the world and drive responsible trustworthy innovation”* (G6 p. 6)  *“It is an opportune moment for G7 nations to lead the way on this conversation, and encourage a consensus on the principles for supporting the development and deployment of AI/ML-enabled medical devices to promote patient safety and foster innovation.”* (G7 p. 5) | WHO (doc 1) ethical principles |
| G8 | ***Definition****:* not available  ***Intended use****: Guiding principles. “The adoption of these principles serves to ensure the safe provision of AI services, and for building trust in the dependability and efficacy of AI in healthcare.****”*** (G8 p. 6) | *“The recommendations in these guidelines are based on principles adapted from the AI guidance provided by the Personal Data Protection Commission (PDPC) and the Monetary Authority of Singapore (MAS).”* (G8 p. 6) |
